# Annotations

**Published:** 1905-02-11

**Authors:** 


### Annotations.

### Industrialism and Infant Mortality.

The doctrine of fatalism is generally regarded as out of harmony with the creeds and economics of Western civilisation, yet both in imperial and municipal policy events are not infrequently described and treated as inevitable. Among these may be placed the view that the ratio of infant mortality must needs be greater in industrial centres than in rural districts. The fact that such actually is the case is beyond question, for figures collected in England show a death-rate in children ranging from fifty to a hundred per cent. in favour of the country as compared with manufacturing towns. But that such a state of matters is a necessity has recently been effectively contradicted by Variot in a paper describing the success which has been obtained at Le Creusot in France. The figures show that the infant mortality for the ten years ending in 1903 has been 110 per 1,000 as compared with 160 per 1,000 for the whole of France, 200 or more per 1,000 in certain French industrial districts, and 180 per 1,000 in English industrial districts. Among the causes for these relatively satisfactory figures great emphasis is placed on the non-employment of women during the period of child-bearing. It is stated that in the 25,000 persons at Le Creusot there are only 18 married women—figures which, in relation to the statistics already quoted, and to the practice common in many other factories, are obviously highly significant. Other conditions in operation at Le Creusot, also doubtless contribute to the low infant mortality. These include the payment of such wages to the men that wives and mothers are not forced to contribute to the support of the household, the provision of gratuitous medical assistance, and the efficient supervision of the housing and hygiene of the workmen and their families. In view of the facts and figures submitted by Variot it would seem unjustifiable and unworthy calmly to acquiesce in the heavy infant mortality which marks so many of our large centres of population.

### Consultations Between Medical Witnesses.

The proposal to establish as a rule of professional ethics and practice the custom that medical men engaged professionally in questions or disputes likely to lead to litigation should meet one another in consultation will certainly be allowed to have an excellent motive. It aims at the disappearance of those unseemly contradictions which occasionally mark the course of trials in the courts of justice, and which have made the name of the expert witness the subject of much scornful wit. In addition, it is hoped that a joint examination and conference, by producing greater accuracy and thoroughness in observation, and a more just appreciation and interpretation of the facts of the case, will favour the settlement of many disputes without the necessity of litigation. If these ends can be attained the gain will be a not inconsiderable one. That such a result is possible is evident from the report of the Medico-Political Committee of the British Medical Association, which announces that the practice suggested has been adopted in Leeds and that members of the legal profession as well as practitioners of medicine are more than satisfied with it. The question has thus passed from the region of mere academic discussion into one of practical demonstration, and it is to be hoped that the good example will be generally followed. There is, however, one aspect of the subject which it is necessary to keep in mind. A medical man instructed to examine an injured person, say, for example, for a railway company, is surely bound not to give his opinion to any person other than those on whose behalf he is acting. To act otherwise would, at least so it seems to us, be a breach of confidence. Hence if the new proposal is to come into practical operation it would be well to intimate to the profession that whilst such consultations are to be desired and ought to be suggested by each medical examiner they should always be arranged with the knowledge and consent of the parties concerned, or of their solicitors. In the report above referred to no reference is made to this point and in this respect we cannot but consider it distinctly defective.

### The Signature of the Prescription.

The physician's prescription, even apart from its therapeutic merits, is a document of considerable authority and influence. Under its instructions the dispenser is justified in delivering to the person possessing it whatever medicines are ordered by the writer. These may even be active poisons, which in ordinary circumstances can only be sold under strict precautions and when duly labelled. Nevertheless, under the magic influence of the prescription all such restrictions disappear, and thus agents of enormous potency may pass into the hands of careless or ignorant persons. In all this there would be little ground for anxiety were the conditions and circumstances of the prescription carefully limited and defined. But, unfortunately, this is not the case. Whatever may be the legal doctrine of property in a prescription, in practice the document and its directions are assumed to be at the disposal of the person for whose benefit it is written. Hence, as is well known, prescriptions are dispensed over and over again without any further consultation with the physician, and copies are often made and presented to the friends of the original patient. This is certainly not in the interests of the public, and there seems to be a consensus of opinion that such a practice ought to be stopped. But an even more serious consideration is the fact that so long as the usual form is observed there is no need for the prescription to possess the authority of a medical practitioner. Any layman with a little ingenuity and trouble may thus obtain a supply of poisonous drugs. One method of checking this would be to replace the present habit of signing a prescription with initials only by a rule that the full name of the physician should be attached. This would at least give the pharmacist an opportunity for inquiry when an unknown name was presented to him. According to the classical authority of Pereira, etiquette demands the full signature when a prescription is written for any member of the Royal Family. Were less distinguished patients treated in the same manner there would be a diminished chance for the layman to obtain powerful drugs either for self-medication or for even more undesirable purposes.

